# The Telemedicine for Patients With Inflammatory Bowel Disease (TELE-IBD) Clinical Trial: Qualitative Assessment of Participants’ Perceptions

**DOI:** 10.2196/14165

**Published:** 2019-06-03

**Authors:** Charlene Connolly Quinn, Sarah Chard, Erin G Roth, J Kevin Eckert, Katharine M Russman, Raymond K Cross

**Affiliations:** 1 University of Maryland School of Medicine Department of Epidemiology and Public Health Baltimore, MD United States; 2 University of Maryland Baltimore County Department of Sociology, Anthropology, and Health Administration and Policy Baltimore, MD United States; 3 University of Maryland School of Medicine Department of Medicine Baltimore, MD United States

**Keywords:** inflammatory bowel diseases, Crohn disease, ulcerative colitis, qualitative research, telemedicine

## Abstract

**Background:**

Inflammatory bowel disease (IBD), comprising Crohn disease and ulcerative colitis, affects 1 to 3 million people in the United States. Telemedicine has shown promise in IBD. The objective of this study, telemedicine for patients with IBD (TELE-IBD), was to compare disease activity and quality of life (QoL) in a 1-year randomized clinical trial of IBD patients receiving telemedicine versus standard care. Treatment groups experienced improvements in disease activity and QoL, but there were no significant differences between groups. Study adherence to the text-based intervention was less than 80%, the targeted goal.

**Objective:**

To understand adherence to remote monitoring, the goal of this qualitative assessment was to obtain TELE-IBD trial participants’ perceptions, including their recommendations for future monitoring.

**Methods:**

In this study, patients attending 3 tertiary referral centers with worsening IBD symptoms in the previous 2 years were eligible for randomization to remote monitoring via SMS text messages (short message service, SMS) every other week, weekly, or standard care. Participants (n=348) were evenly enrolled in the treatment groups, and 259 (74.4%) completed the study. For this study, a purposive sample of adherent (N=15) and nonadherent (N=14) patients was drawn from the TELE-IBD trial population. Adherence was defined as the completion of 80% (278/348) or more of the weekly or every other week self-assessments. Semistructured interviews conducted by phone surveyed (1) the strengths and benefits of TELE-IBD, (2) challenges associated with using TELE-IBD, and (3) how to improve the TELE-IBD intervention. Interviews were recorded, professionally transcribed, and coded based on *a priori* concepts and emergent themes with the aid of ATLAS.ti, version.7 qualitative data analysis software.

**Results:**

Participants' discussions centered on 3 elements of the intervention: (1) self-assessment questions, (2) action plans, and (3) educational messages. Participants also commented on text-based platform, depression and adherence, TELE-IBD system in place of office visit, and their recommendations for future TELE-IBD systems. Adherent and nonadherent participants prefer a flexible system that is personalized, including targeted education messages, and they perceive the intervention as effective in facilitating IBD self-management.

**Conclusions:**

Participants identified clear benefits to the TELE-IBD system, including obtaining a better understanding of the disease process, monitoring their symptoms, and feeling connected to their health care provider. Participants’ perceptions obtained in this qualitative study will assist in improving the TELE-IBD system to be more responsive to patients with IBD.

## Introduction

Inflammatory bowel disease (IBD), comprising Crohn disease (CD) and ulcerative colitis (UC) affects nearly 1 to 3 million people in the United States [1,2]. IBD are chronic diseases characterized by abdominal pain, diarrhea, bloody stools, fatigue, and extraintestinal manifestations [2]. The symptoms have a negative impact on quality of life (QoL) [3] and result in increased health care utilization [4-6]. Effective treatments exist for patients with IBD. However, a significant number of patients have suboptimal outcomes with standard care. Reasons for suboptimal outcomes include nonadherence [5,7], delays initiating treatment, inadequate monitoring, side effects [8,9], poor education [10,11], and lack of access to IBD care [12]. Telemedicine is increasingly being evaluated by health care systems and payers as an alternative service to address deficiencies in health care delivery [13,14].

The objective of this study, telemedicine for patients with IBD (TELE-IBD), was to compare disease activity and QoL in a 1-year randomized clinical trial of IBD patients receiving telemedicine versus standard care. Treatment groups experienced improvements in disease activity and QoL, but there were no significant differences between groups. Study adherence to the text-based intervention was less than 80%, the targeted goal [13]. To understand treatment adherence, the goal of the qualitative assessment reported here was to obtain TELE-IBD trial participants’ perceptions of the system, including their recommendations for future TELE-IBD monitoring.

## Methods

### Study Design, Setting, and Recruitment

#### Parent Randomized Controlled Trial

Study trial protocol and results of the parent randomized clinical study have been previously published [13,15]. Briefly, the TELE-IBD study was a multicenter, randomized, controlled, clinical trial conducted over 12 months. Participants were adults (mean 38.9, SD 12.3 years; 56.6% women) recruited from 3 academic medical center IBD specialty clinics. Participants in the intervention arms completed self-testing with TELE-IBD on either a weekly or every other week basis using a mobile phone–based platform. The system worked on both iPhone operating system and Android operating systems. All but 3 patients used their own phone. A total of 3 patients received a mobile phone from the study for study trial use only.

Participants randomized to the control group received standard care. TELE-IBD was designed using a mobile phone for participants and a decision support server with website for staff and providers. The website provided an interface for staff and providers for participant profiles and collected data from each self-testing session. The provider could individualize alerts and action plans for each participant, if needed. A standard set of baseline action plans based on the criteria were available to providers. If predetermined criteria were met after testing, simultaneous action plans and email alerts were sent to the participant and study team, respectively. The study team reviewed the information and if necessary, consulted the prescribing provider for management changes. Medication changes were updated in the participant profile and communicated to the participant.

Participants in the 2 intervention groups were prompted to respond to a series of SMS text messages (short message service, SMS) grading their IBD symptoms. To assess bowel symptoms and extraintestinal manifestations, abbreviated disease activity forms were used. CD participants completed a modified Harvey Bradshaw Index [16,17]. Overall well-being, abdominal pain, and diarrhea were core questions. UC and indeterminate colitis (IC) participants completed the Simple Clinical Colitis Activity Index [18,19]. Overall well-being, number of bowel movements during the day, and presence of blood in the stool were core questions.

After answering SMS text message questions about their symptoms, participants received a list of medication(s), dose(s), and direction(s). Participants were asked if they experienced any drug side effects. Participants were asked to describe and grade the severity (mild, moderate, or severe) of side effect; moderate to severe side effects generated an alert.

Alerts and action plans were customized for participants and generated based on responses to the core questions in the activity indices for CD, UC, and IC. Depending on the response, participants were assigned to a disease activity zone: the green zone represented patient remission or mild disease activity, the yellow zone represented moderate disease activity, and the red zone represented severe disease activity. Depending on the severity zone, the provider could select an action for the participant to begin.

TELE-IBD intervention participants received an educational tip twice weekly (W group) or every week (EOW group) and at the discretion of the provider. For example, the provider could send an SMS text message during flu season asking participants to obtain a flu vaccination.

### Qualitative Assessment of the Telemedicine for Patients With Inflammatory Bowel Disease Intervention

#### Participants, Design, and Methods

For the descriptive qualitative assessment reported here, the purposive sample of adherent (N=15) and nonadherent (N=14) patients was drawn from the TELE-IBD trial population. To achieve this sample, 50 patients were contacted from each group of adherent and nonadherent patient participants. No patients refused out of disinterest; 1 patient was out of the country, and the remaining patients could not be contacted after multiple attempts. The final sample was not different from those who participated in this qualitative study. Adherence was defined as the completion of 80% or more of the weekly or every other week self-assessments; 3 core questions described above for each IBD condition had to be answered for the self-assessment to be considered adherent. Participants were contacted via telephone; interviewers described the qualitative study and determined participant willingness to participate in this follow-up study. Phone interviews were scheduled at the convenience of participants; the study was described again, and verbal informed consent was obtained. The study Interview Guide is included in the supplementary file as a Multimedia Appendix 1.

### Data Analysis

Qualitative analysis was used to identify themes of participants’ interviews. A codebook of 24 codes was developed based on *a priori* concepts of interest, including symptoms, TELE-IBD core questions, action plans, education messages, and medications and themes that emerged from the SMS text message. Braun and Clarke’s 6 phases of thematic analysis was applied to organize and identify core themes and subthemes [20]. This approach included several steps by the research team including familiarization, professional transcription, generating initial codes, searching for themes, reviewing themes, and finally defining and deciding on meaningful themes. An iterative approach was adopted with the initial coding performed by ER and SC following the completion of the first set of 10 interviews (5 adherent, 5 nonadherent). Subsequent interviews were then conducted and transcribed incrementally with data analysis occurring simultaneously, and new codes were added from the dataset to ATLAS.ti, version 7. ER, SC, and KE reviewed the transcripts and codes during this process until consensus was reached by all members of the research team. Pattern saturation was achieved (and data collection ceased) when no new themes emerged, determined by the point in analysis where no new codes would provide additional value to the identified themes.

A qualitative research team member coded each interview in ATLAS.ti, version 7. A second team member independently reviewed the coding for missed codes or alternative interpretations. Discrepancies were discussed between coders. The process involved sorting and sifting through the narratives to confirm coding and resolve discrepancies in interpretation and meaning. Coded narrative was then compared across the adherent and nonadherent participants. On the basis of comparative analysis, coded narratives were used to develop higher order themes related to their meanings. Team members then reviewed individual transcripts to identify how themes were embedded within the complete interviews. This served to reach consensus on the narrative meaning. Finally, codes were analyzed to determine patterns within and across the adherent and nonadherent participants. Team members also reviewed individual transcripts to identify how themes were embedded within the complete interview. This review served as an important qualitative validity check.

Interviews lasted between 18 to 54 min and examined perceptions of (1) the strengths and benefits of the text-based intervention (TELE-IBD system), (2) challenges associated with using the TELE-IBD, and (3) how to improve the system. The study protocol was reviewed and approved by the Human Research Protection Office at the University of Maryland, Baltimore.

### Measures

Interview guide questions created by study team members were based on (1) the strengths and benefits of TELE-IBD, (2) challenges associated with using TELE-IBD, and (3) how to improve the TELE-IBD intervention (see supplementary materials).

## Results

### Participant Characteristics

Adherent (n=15) and nonadherent (n=14) participants completed interviews (see Table 1). Participants represented patients with both CD (n=16) and UC (n=12) and 1 patient with IC. Both intervention groups were represented, although the majority were participants receiving every other week SMS text messages (69%).

**Table 1:** Demographic characteristics of participants from the telemedicine for patients with inflammatory bowel disease trial participating in qualitative analysis (N=29).

Demographic characteristics		Adherent (n=15), n (%)	Nonadherent (n=14), n (%)	Total (N=29), n (%)
**Disease diagnosis**				
	Crohn	7 (47)	9 (64)	16 (55)
	Ulcerative colitis	7 (47)	5 (36)	12 (41)
	Indeterminate colitis	1 (7)	0 (0)	1 (3)
Female		7 (47)	6 (43)	13 (45)
**Race/ethnicity**				
	Non-Hispanic white	14 (93)	14 (100)	28 (97)
	African American	1 (7)	—^a^	1 (3)
**Protocol**				
	Weekly SMS^b^ text messages	2 (13)	7 (50)	9 (31)
	Every other week SMS text messages	13 (87)	7 (50)	20 (69)

^a^Not applicable.

^b^SMS: short message service.

### Patterns/Themes

The findings include participants’ perceptions of the 3 main components of the TELE-IBD system: (1) self-assessment questions, (2) action plans, and (3) educational messages. The main themes identified by participants are summarized in Table 2. We also addressed specific questions posed by the University of Maryland School of Medicine TELE-IBD team (RC, KR, CQ) regarding text-based platform, depression and adherence, TELE-IBD system in place of office visit, TELE-IBD perceptions, and participants’ recommendations for future TELE-IBD systems.

**Table 2:** Summary of themes from qualitative interview of patients from the telemedicine for patients with inflammatory bowel disease trial.

Theme	Strengths	Weaknesses
TELE-IBD^a^ self-assessment questions	Improved awareness of symptoms and health status Two-way connection with the treatment team Creates digital log of symptoms and weight Easy to respond	Repetitive when asymptomatic Unwanted or ill-timed reminder of disease Relevance when experiencing atypical symptoms Source of alarm regarding potential symptoms Risk of fixating on weight
TELE-IBD action plans	Reminder to maintain routine, take medications Prompted calls from the treatment team	Accuracy of zone rating—unable to provide context for responses or correct data entry errors Unable to indicate call back is not needed
TELE-IBD educational messages	Improved health literacy: (1) understanding of the disease process and treatment and (2) vocabulary for communicating with treatment team Provides information to share across social network Similar to a trivia game	Repetitive over time Not tailored to specific disease type Not helpful for those already well-informed
Text platform	Convenient Ease of connection with provider	Response window/timed lockouts Cell phone provider problems Inconvenient timing of messages No opportunities to elaborate or correct responses

^a^TELE-IBD: telemedicine for patients with inflammatory bowel disease.

### Self-Assessment Questions

Both adherent and nonadherent participants had positive and negative perceptions of the core assessment questions. In terms of the benefits of the core assessment questions, participants suggest the assessments improved their awareness of their symptoms and health status. Numerous examples of this perception are found across adherent and nonadherent participants:

It was a beneficial kind of reminder to, you know, like “hey, let me think about this - Ah, I’m feeling good.”

Nonadherent participant

I actually kind of liked having to do it because it was kind of like, you know, bam another good week, bam another good week. It was almost like setting personal records or something.

Nonadherent participant

All the questions that they asked seemed like they actually pertained to things that all I had, I could possibly have going on, and I might actually say “yes” to a lot of them...it would kind of give me a little heads up I guess if I should definitely go ahead and schedule an appointment to get in there to see them.

Adherent participant

It inspired me to...keep an eye on my weight and so really pay attention to what was going on and not wait until I was in fold over pain.

Adherent participant

Participants also reported that the assessment questions created and sustained an important 2-way connection with their health care provider. Through remote monitoring, the patient had an efficient, ongoing means for alerting the provider to problems. Simultaneously, the provider could alert the patient if the symptoms were cause for concern. Knowing that the provider’s office was receiving and reviewing participants’ responses was reassuring. A total of 1 participant described there was peace of mind in “monitoration.” Participants also appreciated that their responses formed a log that the provider could then review and discuss. Flare-ups and weight changes that occurred between scheduled office visits were documented, providing a more comprehensive portrait of the participant’s disease experience.

Participants across the sample viewed the assessment questions as straight-forward and easy to answer. Participants did not report embarrassment in recording their responses. The general consensus across the sample was that weekly assessments worked well, but as is discussed next, entering regular assessments when one is feeling well can be tedious.

Critiques of the assessment questions often revolved around perceptions that the questions were (1) repetitive, particularly when not in a flare or (2) “generic,” that is, did not address symptoms that the participant experienced. Both adherent and nonadherent participants raised these concerns. For example, regarding the tedious nature of the assessments, adherent and nonadherent participants described:

When you’re not in a flare and these messages keep coming and it’s the same thing, and you’re like, ‘oh, God, I’m answering the exact same questions over and over and over again. That’s when, you know, like after like tenth time, you’re just like, ‘gee.’

Adherent participant

Sometimes I felt like I got too many texts, I was like, “I’m okay!” you know. But I would reply.

Nonadherent participant

The assessment questions also at times could be “scary” if the participant had not encountered a specific symptom (eg, questions about fistulas) or served as unwanted reminders of one’s disease during a period of remission.

Many believed the content of questions were appropriate. However, for participants who had unusual symptoms or other autoimmune disorders, the questions did not seem relevant. These participants indicated they would have preferred more individualized assessment questions.

Participants’ conflicting attitudes toward tracking their weight also should be recognized. Although many participants perceived regular weigh-ins as a helpful, independent indication of their disease status, others were concerned about fixating on the scale:

It’s one of those things where I think it was helpful and I think it’s a really good thing for the doctor to see but sometimes I don’t like weighing myself. Right now I don’t keep a scale in my house because I don’t really necessarily want to know the number. I can tell when I’m losing weight and I don’t really like obsessing about that number. I know for medical purposes you’re going to want to track things that have a numerical value but I mean I can tell by the way I feel and how my clothes are fitting and how I look if I’ve been losing weight or anything...

Adherent participant

Finally, even if participants critiqued the core assessment questions, they perceived that the assessment process could be helpful for others:

I’m going to be on top of it whether I have this text messaging or not. [...] It’s not going to help all. Some of them, it’s not going to matter, but I believe it will help some of them. It will push some of them along, you know, to go and get the proper treatment and not delay, and to be honest with your doctor about your symptoms and everything.

Adherent participant

### Action Plans

Participants had difficulty recollecting the action plans, often needing reminders from interviewers that the system provided a green, yellow, or red zone indicator at the end of the assessment. Although appreciative of the reminder to take their medication or to continue with their routines, participants who were mainly in the green zone did not remember receiving an action plan.

Those who did recollect receiving yellow or red indicators when they were not feeling well, which resulted in a call-back from the providers’ nurse staff, were often appreciative of the ease and immediacy of the contact with the provider’s office. As 1 adherent participant described, knowing symptoms would produce a TELE-IBD action plan was helpful:

I knew, OK if it was Wednesday, I know I’m getting the call Thursday so I would just wait and do that instead of calling the nurse and saying, “Hey this is what’s going on.” If I knew it was something that would put me in the yellow zone, I would just wait...

Adherent participant

I really liked it believe it or not because I was able to provide feedback on a weekly basis and I was often, depending on my answers, getting calls immediately practically from the doctor or nurse. She’s like, “what’s going on?”...OK, I’ll talk to the doctor and we’ll do whatever if need be. I thought that was awesome.

Nonadherent participant

The primary critique of the action plans was that the zone ratings were not perceived as accurate, and participants felt they had no means to provide explanatory information. Participants indicated, for example, that at times their assessments and subsequent zone ratings reflected other illnesses, life events, or even data entry errors. Rather than receiving the call from the provider’s office, participants would have liked to have been able to indicate why no follow-up was needed.

### Educational Messages

Participants described mixed perceptions of the educational messages. Enthusiasm for the educational messages did not vary by adherence status or whether the participant was in remission at baseline.

Participants who liked the educational messages appreciated learning more about the disease, the intestinal system, diet strategies, and terminology that could then be used when talking with their health care provider. Participants who liked the messages described them as “enlightening,” “kinda fun,” and helpful “trivia.” To these participants, the educational messages reinforced their health literacy; as one described:

I got all these things in the back of my mind that hey, this could be something, you know, it just makes it easier to, you know, catch that in the future.

Adherent participant

Another indicated the messages served as important refreshers, or “a ha” moments that helped her more quickly identify symptoms:

I was getting a lot of little snippets of information, parts of information I already knew but maybe forgot about so when I got the information it was like oh yeah, oh that’s right. So it was helpful in that sense and it helped me, I feel like I’m in more control of my body. I know when I’m getting the symptoms. I know when something starts to irritate me a lot quicker than I did in the past.

Adherent participant

Disease information then traveled along participants’ social networks, as participants indicated they would share messages with spouses or other family members with similar conditions.

The most common negative comment on educational messages was that over time the messages became repetitive. Participants recommended reducing the repetition and adding more information on vitamins, probiotics, or diet. Others commented that the messages were not relevant to their disease. For participants who had a long history with their illness or a strong understanding of its triggers, the education messages did not provide new insights, such as this nonadherent participant:

I felt that they were kind of generic, but I also had been diagnosed and had been dealing with the UC for, you know, probably over five years at that point.

Participants who worked in an allied health field also felt the messages were not informative. It is important to note that participants who critiqued the educational messages commonly indicated that they believed the messages could be of great value to others. As a nonadherent participant with a clinical background suggested, “I can understand how it would be helpful for someone who was coming at it from a different perspective.”

### Platform: Text-Based System

Participants generally approved of the mobile phone platform for the text-based telemedicine system. Among the participants for whom texting is a part of daily life, the system was a “short” and “convenient” way to monitor their symptoms and stay connected to their provider. Participants who did not like the mobile system objected to specific design features or noted problems attributed to their carrier.

Regarding design features, the chief design criticism was that data entry errors could not be corrected, and there was no means to communicate errors to prevent a call-back from their provider. Participants also perceived that the numerical response system did not fully capture their health status and were frustrated that there was not space in the system to elaborate on their current situation. Participants further noted that the timing of the SMS text messages became inconvenient over the course of the study if their work or family responsibilities changed.

Technical issues such as delayed responses from the TELE-IBD system were described as “extremely irritating,” significantly extending the amount of time required to complete the assessment. Participants perceived the response window as too short to accommodate the vagaries of life and issues caused by their cell phone providers. As 1 adherent participant described:

It wasn’t necessarily inconvenient, but there were times that I would see that I got the messages and it was like a time sensitive, if you didn’t respond, you know, it wouldn’t prompt the next questions. So there were times that I missed like that particular like set of questions just because it, you know, it like timed out or I wasn’t able to finish answering because I was in class or busy or doing whatever.

Participants who were not satisfied with the delivery mechanism noted that having an option for email delivery or some other type of survey delivery would be helpful. However, this should not be interpreted as a recommendation to switch entirely to an email system as others felt strongly that current delivery method was effective.

### Depression or Mood Effect

The UM-SOM TELE-IBD team (RC, KR, CQ) found that depression correlates with adherence, especially among younger participants [21]. One 20-year-old participant (nonadherent) is a good example of this finding. He perceived the messages as a lifeline. He said he struggled to find people to talk to who would understand what he was going through:

When I would get these messages, it made me feel better because it made me realize that I’m not actually the only one that had these kinds of problems. So, when I would get them, I would get a bit excited, a good bit excited and I would feel relieved. Like a ten-pound weight had been lifted off my shoulders or off my chest.

In this study, emotions were tied closely to one’s health status. When in a flare, participants reported feeling stressed, depressed, or anxious. These participants were more likely than those who were not in a flare to be adherent to the TELE-IBD system core self-assessment questions. An adherent participant described what it was like to be in a flare, addressing indirectly how responding to the TELE-IBD system may have been part of the internal focus on one’s disease:

Well when I was in the flare-up, it was, I’m more, what’s the word, introverted, you don’t talk as much, you’re very concerned about what’s going on in your body, so you don’t focus on much more than that.

A nonadherent participant likewise eventually reasoned that feeling ill might lead one toward being more responsive:

I do suppose if, if someone were really feeling down about it, it could feel like one more burden of oh goodness why am I doing this. But on the other hand, if they’re having issues like that then that’s all the more reason to be in communication with their physician.

We also asked participants to reflect on the finding regarding depression. Several expressed surprise that being depressed is associated with adherence. A nonadherent participant responded by saying, “Ah!” and then, “Yeah, well it’s, it does feel like, you know, someone’s asking you how you’re doing and it’s a way to engage you for this from afar so I can definitely see that.” Another nonadherent participant could see how this might be: “I think when people feel poorly, they’re probably happy to answer as many questions as it’s going to take for them to get helpful information.” At the same time, an adherent participant cautioned that receiving TELE-IBD SMS text messages while being depressed might make someone more depressed:

If somebody was more depressed, I definitely think it would have affected them differently but I’m not sure if would be positive or negative. Maybe more negative because it’s a constant reminder that you kind of have the disease.

Finally, participants also indicated that the emotional impact of the disease was not well-incorporated into the system, for example, the core self-assessment questions. One participant explained:

I would say, and this isn’t just for the study but in general, I think a bigger focus on emotional impact of the disease and also the fatigue. I think a lot times you focus on, you know, weight and, and pain levels and other things but a lot of it is just, it’s very fatiguing and, you know, there’s a lot of malnutrition, malabsorption that goes on. So I think fatigue is just one, is, you know, it’s one of the more debilitating aspects and it’s surprisingly so, I think people focus on, it must have hurt, you know, stomach pain all the time but you kind of build a tolerance to that but it’s hard to build a tolerance to being tired all the time.

Adherent participant

### Impact on Face-to-Face Office Visits or Other Forms of Clinical Care

Participants had mixed perceptions on how participating in a TELE-IBD system would impact their routine health care. Some indicated that if they were not having any IBD-related issues, checking in through the TELE-IBD system could substitute for a face-to-face visit. This was an especially attractive concept for participants who travel a long distance for provider office visits. However, others expressed concern that they would still want to meet with their provider twice a year or in the event of a flare. For example, 1 adherent participant who lived an hour from her provider expressed the system could help monitor, but:

I would still want to go in and make sure my medication is OK...Over the six months a lot of times I might develop like a question that I might want to ask and through that, you know, the text message you’re not able to necessarily do that. So, it’s nice to be able to go in and have that dialogue about certain things that I might be experiencing.

### Adherence, Health Status, and Telemedicine for Patients With Inflammatory Bowel Disease Perceptions

A few patterns emerged regarding adherence, participants’ perceptions of the relevance of the system, and experiences with design and technical glitches.

First, regarding the relevance of the system, nonadherent participants were much more assertive in indicating they actively managed their disease and/or had a strong understanding of the disease (whether because of their own research, the length of time diagnosed, or their professional background), such that the system was less helpful to them than it may be to others.

Second, although both adherent and nonadherent participants experienced technical difficulties, discussions of the technical problems were much more salient among the nonadherent participants. The system became “extremely irritating” when the SMS text message questions timed out too quickly, extending the time required to complete the questions to 15 min or more.

Regarding the role of health status, both the adherent and nonadherent participants noted the value of the TELE-IBD system during a flare or when changing medications. The TELE-IBD system was clearly recognized as allowing for a more immediate and effortless way to communicate with the doctor’s office. It also is very reassuring to receive the doctor’s call back when experiencing symptoms. Although both groups reported the system was “repetitive,” the nonadherent tended to voice more strongly that the regular pings from the system were “annoying” when they were feeling well.

## Discussion

### Principal Findings

To our knowledge, this is the first telemedicine for IBD post-RCT qualitative study of adherent and nonadherent intervention participants. The main finding was that patients prefer a flexible follow-up system that is personalized, including education messages that can be targeted to individual patients and decreased repetition. A telemedicine system that is 2-way communication, easily connecting patients with providers and provides “actionable” responses to patient self-assessment is preferred. Telemedicine implemented as an adjunct to patient-initiated consultations and self-management and regular office visits was identified as optimal approaches to meet the patients’ needs. The TELE-IBD system was reported by participants as effective in facilitating IBD self-management. Participants also considered the TELE-IBD useful in treatment decisions made with providers in-between office visits. New models of IBD telemedicine care could improve the patients’ experience of care and reduce or delay unnecessary health care utilization.

The TELE-IBD intervention in this study followed a participatory care framework by testing pilot feasibility [22,23] and acceptability, followed by a randomized clinical trial [13,15] and follow-up qualitative evaluation of adherence and nonadherence to the text-based intervention [24]. Evaluating performance at each of these phases is critical to the overall success of the intervention and to ensure that the system is safe and beneficial to patients. Although adherence in this study was targeted to 80% (actual=60%), this qualitative assessment demonstrates ongoing patient engagement to help mitigate problems associated with risk of attrition when evaluating new models of care such as telemedicine.

### Limitations

We recognize the reported study has limitations. The qualitative assessments were from patients willing to be interviewed and may not be representative of all patients in the TELE-IBD clinical trial or other IBD patients considering a telemedicine system of care. We attempted to address this by recruiting adherent and nonadherent persons to be interviewed. Interviews occurred after use of the system. Memory or recall may have affected participants’ responses. Finally, it’s important to note that although clinicians and scientists have expectations for adherence, not all patients want to be “engaged” all the time. Patient engagement is a continuous behavior, and our assessment of adherence may have captured patient perceptions at a single point in time.

### Conclusions

Participants, both adherent and nonadherent, identified clear benefits to the TELE-IBD system, including obtaining a better understanding of the disease process, monitoring their symptoms, and feeling connected to their health care provider. In this sense, TELE-IBD facilitates access to care, particularly for those who live a long distance from the provider and/or are reluctant to or have difficulty contacting the provider between scheduled office visits. Both educational messages and core assessment questions contribute to these perceptions of the TELE-IBD system. Such a connection is particularly welcome during an IBD flare and not as necessary when asymptomatic.

Participants experienced considerable recall issues around the action plans, although could remember the color zones once prompted and appreciated knowing that they would receive a provider call if experiencing critical symptoms. Reminders regarding medication were appreciated by some, but others said the reminders were not necessary. The study reported here demonstrates the importance of qualitative assessment of participants’ views in technology-based interventions. On the basis of our results, future studies of revised remote monitoring systems in patients with IBD are needed. To promote patient engagement, future systems should include flexible options for testing (mode of testing and frequency of testing), reduce “irritant” factors such as timed lock outs and set testing schedules, and allow for corrections in responses. Furthermore, assessment of symptoms and receipt of action plans and educational messages should be as personalized as possible. Finally, remote monitoring should remain an adjunct to in-person monitoring.

## Appendix

Multimedia Appendix 1 Study interview guide.

## Abbreviations

CD: Crohn disease
IBD: inflammatory bowel disease
IC: indeterminate colitis
QoL: quality of life
SMS: short message service
TELE-IBD: telemedicine for patients with inflammatory bowel disease
UC: ulcerative colitis
