# Supplementary material for: The Telemedicine for Patients With Inflammatory Bowel Disease (TELE-IBD) Clinical Trial: Qualitative Assessment of Participants’ Perceptions
Source: J Med Internet Res. 2019 Jun 3;21(6):e14165. doi: 10.2196/14165 (PMC6746080; doi:10.2196/14165)
Supplement: Multimedia Appendix 1 [file jmir_v21i6e14165_app1.docx]

***TELE-IBD program-Qualitative Interview Questions***

1. *Tell us about your experience with the TELE-IBD program…*
2. *There are many symptoms associated with IBD; What types of symptoms do you experience with your IBD? [probe frequency, duration]*
   1. *Which symptoms are of most concern to you? [probe why, e.g., health/social]*
   2. *What do you typically do when you experience these symptoms? [probe: changes in activities; diet; contact provider]*
   3. *Which symptoms are not as concerning? [probe why; what do you do for these symptoms]*
3. *Did you use the text message (TELE-IBD) program to report symptoms?*
   1. *Are there some symptoms that you would generally try to report? Follow up: are there some you would not? Probe for why report/not report certain symptoms*
   2. *Were the responses through the text system helpful? [probe how/why/why not]*
   3. *Are there ways the system could be more helpful?*
   4. *Thinking of any times when you did not report symptoms, what were some of the reasons? [probe for issues related either reporting or not reporting and reasons, for example, convenience/inconvenience, new symptoms, repetitive symptoms, fear, privacy, social context, emotional state (feeling down or overwhelmed)]*
4. *Did you have any problems using the TELE-IBD system? [probe for description, what did participant do, how resolved; be sure to ask about problems with the phone, texting, connectivity]*
5. *How did you feel about texting information about your symptoms? Your weight? [Probes for each: privacy, embarrassment, time involved]*
6. *Did the TELE-IBD system help you manage your symptoms? [describe how; if action plan is mentioned use probe for #7 below]*
7. *Did you receive an action plan through the TELE-IBD system? Tell me about your action plan. Probes: [if discussed with #6, probe for additional plans]*
   1. *Was it helpful to have an action plan(s)?*
   2. *Were you able to implement the action plan? [describe]*
   3. *Did you encounter challenges with your action plan(s)? [describe]*
   4. *Did your use of the action plan(s) change over time? [describe]*
8. *If not mentioned above, did the TELE-IBD program help you manage your medications? [describe]*
9. *Do you experience side effects from your medications? [describe]*
   1. *What do you typically do for the side effects?*
   2. *Did the program help you in managing your side effects? [describe]*
10. *Did you read the education messages that you received? [probe why/why not]*
    1. *Where they helpful? [describe why/why not]*
    2. *How could they be more helpful?*
11. *Did your use of the TELE-IBD program change over time? [probe: as symptoms became more/less severe]*
12. *What did you like about the TELE-IBD program? [probe for which/why]*
    1. *Thinking of all the components of the TELE-IBD program, which were most helpful? (probe why)*
13. *What did you dislike about the TELE-IBD program? [probe for why]*
14. *Are there ways to change the TELE-IBD program to make it more helpful?*
    1. *Probes: content; frequency; platform*
    2. *Similarly, are there ways to make it easier to use?*
15. *How would you describe your overall health?*
    1. *How would you describe your quality of life?*
    2. *Did the TELE-IBD system impact your quality of life? (probe how: positive/negative)*

©UMBC/UMB TELE-IBD STUDY.
